# Morphometric Study of Proximal End of the Fully Ossified Human Femur: A Cross-Sectional Study

**DOI:** 10.7759/cureus.29188

**Published:** 2022-09-15

**Authors:** Sampada V Late, Harsha Keche

**Affiliations:** 1 Medical Anatomy, Government Hospital Samudrapur, Wardha, IND; 2 Anatomy, Jawaharlal Nehru Medical College, Datta Meghe Institute of Medical Sciences (DU), Wardha, IND

**Keywords:** length of an intertrochanteric crest, neck-shaft angle, neck length, circumference of the neck, transverse diameter, vertical diameter, circumference of head, proximal breadth, femur

## Abstract

Introduction

The femur or thigh bone is the longest and strongest bone in the body. It provides skeletal support for the thigh. Weight-bearing and stability of gait are the essential functions of the femur.

Aim

To construct the baseline data on different dimensions of the proximal end of the fully ossified human femur and determine any significant differences between the right and left femora.

Objective

To measure the total length of the femur, proximal breadth, vertical diameter of the head, transverse diameter of the head, the circumference of the head, the vertical diameter of the neck, the transverse diameter of the neck, the circumference of the neck, anterior length of the neck, neck-shaft angle, length of the intertrochanteric crest and correlate the different dimensions of the proximal end with the total length of the femur.

Result

The variables of the proximal end of the left femora like proximal breadth (PB), the circumference of the head (CH), vertical diameter (VDN), the transverse diameter of the neck (TDN), the circumference of the neck (CN), neck length (NL), neck-shaft angle (NSA), length of an intertrochanteric crest (LITC) showed highly significant positive linear correlation with the length of the femur. Circumference of the neck showed the highest degree of correlation with the length of the femur (correlation coefficient 0.839).

Conclusion

The statistically significant difference between right and left femora was found between the length of the femur, the vertical diameter of the neck of the femur, the transverse diameter of the neck of the femur, and the length of the intertrochanteric crest of the femur. The mean value of proximal breadth, the vertical diameter of the head, the transverse diameter of the head, the circumference of the head, the circumference of the neck, anterior length of the neck of the femur, and neck-shaft angle of the right and left femora to have no statistically significant differences.

## Introduction

The roots of the term "femur" are "fero" (to bear) and "foetus" (to be born). In English, the phrase "thigh bone," which is an architectural term, was first used to describe the femur in 1799. It has proximal and distal ends, as well as a shaft. An articular surface connects the spherical head of the femur and the acetabulum of the pelvic bone [1,2] A roughened depression known as the fovea serves as the anchor for the circular ligament of the femoral head, which is located somewhat below and beyond the head's center [3].

The head of the femoral shaft is connected to the long axis by the 125-degree femoral neck, a cylindrical bone strut that is roughly 5 cm long [3]. This angle is referred to by a number of names, including neck-shaft angle (NSA), collo-diaphyseal angle (CDA), diaphysis-femoral neck angle, angle of the neck of femur, angle of inclination, cervicodiaphyseal angle, and collum diaphyseal angle [4] The femoral shaft must be capable of swinging free of the pelvis in order for the movement to occur [5]. The cervicodiaphyseal angle is larger at birth, measuring an average of 160, but as the skeleton grows, the angle shrinks, and in adulthood, it only reaches an aggregate of 135 (or less). There was hardly any appreciable distinction between the two sexes; however, the CDA on this right side is typically lower than the one on the left. The implants are employed to cure proximal femur fractures, including the 135 CDA, according to measurements taken in Caucasians. Consequently, a reference value of roughly 135 is employed in the production of implants for Caucasians who undergo orthopedic surgery [6].

Researchers employ a variety of techniques to gauge the femur's size. Mechanical measurements of the femur's dimensions are made on cadaveric bones; however, patients often undergo imaging tests such as roentgenograms, CT scans, MRIs, and ultrasounds [5] These investigations demonstrate that the findings of western studies are not comparable to the Indian population since the measures of the femora vary among both communities [7]. The investigation of the morphometric features of the proximal femur in dry bones, as well as the integration and consistency of data for a particular population, is a key principle for the determination of risk factors in pathological situations, preoperative planning, and for the creation of prosthetic components [8].

## Materials and methods

Source of data

The present study was performed in the Department of Anatomy, Jawaharlal Nehru Medical College, Datta Meghe Institute of Medical Sciences, Sawangi (Meghe), Wardha, on 200 dry femora (100 right femurs and 100 left femurs) in two years.

Study design and sample size

A cross-sectional study was used in this study. A total of 200 human adult femora (100 right and 100 left) are used for the present study.

Study Setting

The present study was carried out in the Department of Anatomy, Jawaharlal Nehru Medical College, Datta Meghe Institute of Medical Science (Deemed to be University), Sawangi (M), Wardha, India.

Inclusion criteria

Fully ossified dry femurs from human corpses of both sides of either sex are used for our study.

Exclusion criteria

Any femur showed visual osseous pathologies like tumors, deformities, fracture, trauma, and a significant malformation, abnormality, or damage that could affect its shape and structure.

Instruments and measurements

**Figure 1:**
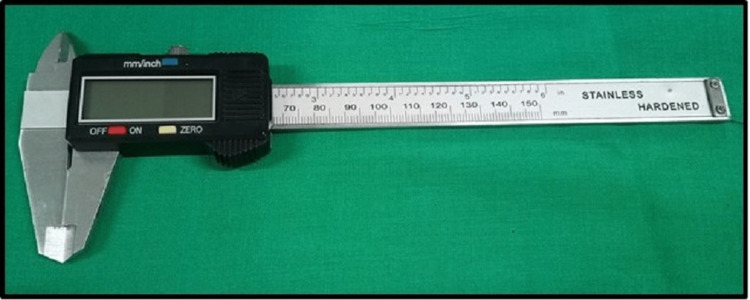
Digital Vernier Caliper

**Figure 2:**
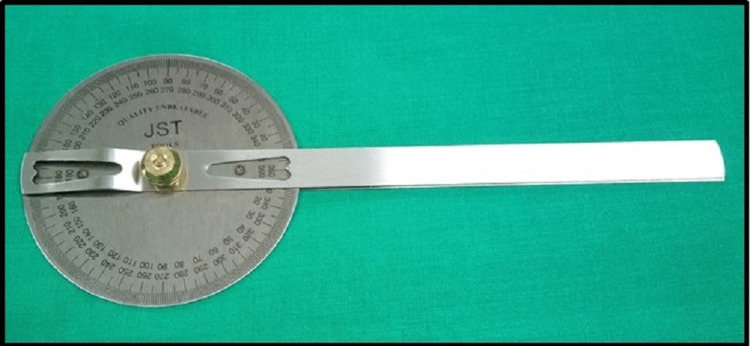
Goniometer

**Figure 3:**
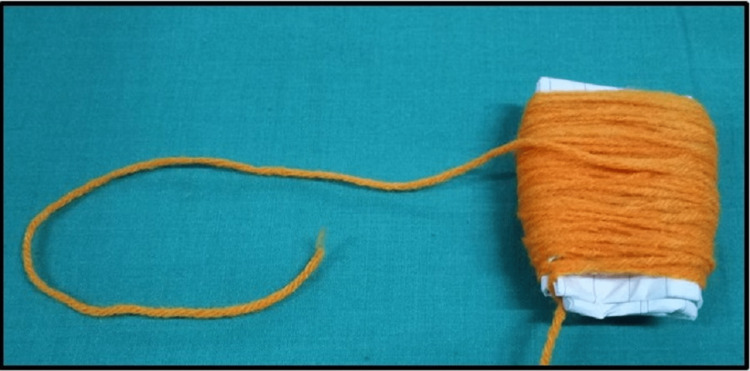
Colored Thread

**Figure 4:**
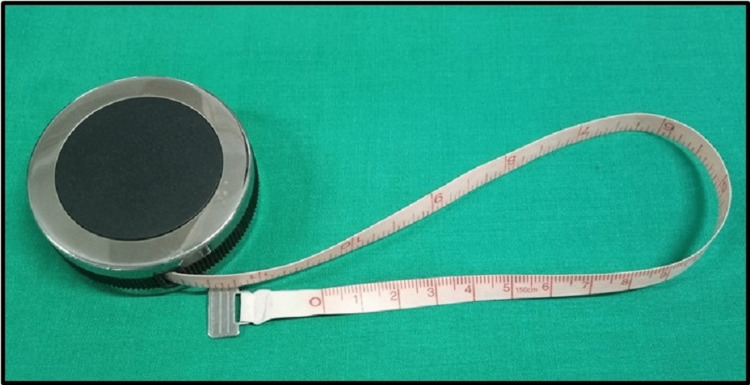
Measuring Tape

The instruments used for measuring various parameters of femora are a digital vernier caliper, a goniometer, colored thread, and a measuring tape (Figure 1-4).

Statistical Analysis

The statistical analysis was done by the SPSS (IBM Corp. Released 2015. IBM SPSS Statistics for Windows, Version 23.0. Armonk, NY: IBM Corp). The Pearsons correlations method was applied to test the difference between the right and left femur.

Ethical consideration

The ethical approval was reviewed and approved by the Datta Meghe Institute Medical Sciences (DMIMS)(DU)/IEC/Dec-2019/8040.

## Results

In this research study, the bones are distributed into 100 on each side to identify the difference between the femur of the right and left sides (Table 1).

**Table 1:** Difference between the femur length of the right side and left side. Non-Significant (NS), Significant (S)

Length of femur	Right Side		Left side		t-Value	P-value
	Mean	SD	Mean	SD		
	42.32	3.84	41.46	1.23	2.151	0.033 S (p value ˂ 0.05)
Proximal breadth (PB) (cm)	85.13	3.78	84.37	6.90	0.965	0.336 NS (p-value ˂ 0.05)
Vertical Diameter of Head (VDH) (mm)	40.89	2.05	41.46	2.77	1.651	0.100 NS (p-value ˂0.05)
Transverse diameter of Head (TDH)(mm)	41.05	2.15	41.54	2.69	1.41	0.158 NS (p-value ˂ 0.05)
Circumference of the head (CH) (cm)	13.14	0.49	13.32	1.12	1.505	0.134 NS (p-value ˂0.05)
Vertical Diameter of Neck of the Femur (VDN) (mm)	28.45	4.22	27.43	2.20	2.141	0.034 NS (p-value ˂ 0.05)
Transverse Diameter of Neck (TDN) (mm)	22.33	1.24	23.25	3.22	2.657	0.009 S (p-value ˂0.05)
Circumference of the neck (CN) (cm)	9.73	7.93	8.93	0.43	1.012	0.313 NS (p-value ˂0.05)
Length of the neck (NL) (cm)	2.86	0.36	4	12.02	0.946	0.345 NS (p-value ˂0.05)
Neck Shaft Angle (NSA) (^0^)	119.29	3.34	121.16	9.91	1.787	0.076 NS (p-value ˂ 0.05)
Length of Intertrochanteric Crest (LITC) (cm)	5.92	0.52	6.20	0.87	2.804	0.006 S (p value ˂0.05)

The dimensions of the proximal segment of the right femora were measured. The total length of the femur (TFL) ranged from 39.50 cm to 43 cm, with a mean of 41.46 cm, and a standard deviation is 1.23 cm. The variables of the proximal end of the right femur, i.e., proximal breadth (PB), circumference of head (CH), vertical diameter of head (VDN), transverse diameter of head (TDN), circumference of neck (CN), anterior length of the neck (NL), neck shaft angle (NSA) and length of intertrochanteric crest (LITC) showed a highly significant positive linear correlation with the total length of the femur. Among the variables, the vertical diameter of the neck showed the highest degree of correlation with the total length of the femur (correlation coefficient 0.694) (Table 2).

**Table 2:** Correlation of the different dimensions of the right femur with the TFL. Non-Significant (NS), Significant (S)

Measurement (Right femur)	Minimum	Maximum	Mean	SD	Correlation with total femoral length	P-value
Total length of the femur (TFL)	39.50	43	41.46	1.23	-	-
Proximal breadth (PB)	81	94	85.13	3.78	0.340	0.0001S
Vertical diameter of head (VDH)	38	44	40.89	2.04	0.635	0.019NS
Transverse diameter of head (TDH)	39	45	41.05	2.15	0.581	0.100NS
Circumference of head (CH)	12.60	14	13.14	0.49	0.000	0.0001S
Vertical diameter of neck (VDN)	24	32	27.43	2.20	0.694	0.0001S
Transverse diameter of neck (TDN)	20	25	22.33	1.24	0.641	0.0001S
Circumference of neck (CN)	8	9.50	8.93	0.43	0.003	0.0001S
Anterior length of the neck (NL)	5	6.50	5.92	0.52	0.140	0.0001S
Neck shaft angle (NSA)	112	124	119.29	3.34	0.419	0.0001S
Length of intertrochanteric crest (LITC)	2.50	3.40	2.86	0.36	0.000	0.0001S

The dimensions of the proximal segment of the left femora were measured. The total length of the left femur ranged from 39.50 cm to 45.50 cm, with a mean of 42.62 cm, and a standard deviation is 1.90 cm (Table 3).

**Table 3:** Correlation of the different dimensions of the left femur with the TFL. Non-Significant (NS), Significant (S)

Measurement (Left femur)	Minimum	Maximum	Mean	SD	Correlation with total femoral length	P-value
Total length of the femur (TFL)	39.50	45.50	42.62	1.90	-	-
Proximal breadth (PB)	44	93	84.37	6.90	0.000	0.0001S
Vertical diameter of head (VDH)	35	44	41.46	2.77	0.373	0.069NS
Transverse diameter of head (TDH)	36	44	41.50	2.69	0.428	0.095NS
Circumference of head (CH)	11.50	14	13.25	0.82	0.000	0.0001S
Vertical diameter of neck (VDN)	24	33	28.10	2.78	0.025	0.0001S
Transverse diameter of neck (TDN)	19	43	23.25	3.22	0.000	0.0001S
Circumference of neck (CN)	8	10	8.93	0.65	0.839	0.0001S
Anterior length of the neck (NL)	5.50	7	6.13	0.47	0.420	0.0001S
Neck shaft angle (NSA)	115	130	122.06	4.36	0.000	0.0001S
Length of intertrochanteric crest (LITC)	2.50	3.50	2.79	0.40	0.00	0.0001S

## Discussion

The current study, titled "Morphometric examination of the proximal end of the fully ossified human femur," sought to establish preliminary data on several measurements of the proximal end of the femur and determine whether there are any significant differences between right- and left-sided femur bones. Two hundred dry femora-one hundred on the right side and one hundred on the left-made up the study material. The purpose of this study was to compare the morphometry of the right and left sides of the proximal end of the femur in order to create orthopedic implants for the treatment of femur fractures. Injury occurrences like femur neck fractures are becoming more common. Implants are being used to correct those fractures in accordance with the proximal femur's dimensions.

Researchers worldwide have been using numerous methods to measure bones over the last few decades. The bones were measured mechanically by ultrasound, computerized tomography (CT), roentgenography, and magnetic resonance imaging (MRI) on the cadaveric bones and patients. The framework of this research has found that assessments differ in population and procedures used. The mean total lengths of the right femur were found to be 42.32 ± 3.84 cm, and accordingly of the left femur was 41.46±1.23cm, respectively, and its difference was identified to be statistically significant. In the study conducted by Ziylan et al. [9], right and left femur lengths were 41.68 cm and 42.84 cm, respectively, which concur with our study. Ravi et al. [10] recorded a total femoral length of 447.9 mm and 446.2 mm of the right and left femurs, respectively, higher than our study. According to Khalil et al. [11], the greatest length of the femur is the most accurate criterion for classifying unknown femora. Typically, the male femur is bigger than the female femur. The sex distinction component for long bones states that male bones are generally longer and larger than female bones [11,12].

The mean proximal breadth of the femora on the right side was found to be 85.13±3.78 mm, and accordingly of the left side was 84.37±6.90 mm mm, and their difference was then found to be statistically non-significant. The proximal breadth on the right side was 86.1±4.2 mm, and that on the left side was 85.8±5.2 mm, in the study of the metric assessment of femur in the Brazilian community conducted by De Sousa et al. [6], this is by our study while other researchers found higher values. Ziylan et al.[9] found 90.1mm and 90.2mm proximal breadth of the right and left femur, respectively, higher than our study. Sreekumar [13] found that the proximal breadth of the right femur was 78.0 mm, and that of the left femur was 79.04 mm, which is lower than the present study.

Dwivedi et al. [14] found that the mean vertical diameter of the right femur was 40.57±3.54 mm and of the left femur was 40.49±3.49 mm. Silva et al. (2003) [12], in their study, concluded that the average vertical diameter of the head of the right femur was 41.63±3.20 mm and of the left femur was 42.96±3.59 mm. Both studies are in concurrence with our study. Kamdi et al. [15] recorded a higher vertical diameter, while Sreekumar [13] recorded a lower vertical diameter than our study. Purkait [16] identified that the vertical head diameter of the right femur was significantly more significant than that of the left [15].

In the osteometric study of a human femur, Khaleel et al. [10] stated that the femur's vertical diameter gave a higher accuracy percentage sexing the femur. Pearson presented data on mathematic sexing of the femur and concluded that for sexing of the femur, the vertical diameter of the head is one of the criteria; Pons also gave the same opinion [10].

The present work shows that the mean transverse diameter of right-sided femur bones was 41.05±2.15 mm, and that of the left side was 41.54±2.69 respectively, and was found to be statistically non-significant. Dwivedi et al. [14] found that the mean transverse diameter of the right femur was 40.59±3.47 mm, and of the left femur was 40.30±3.48 mm. Rashid et al. [17] investigated the morphometry of the femur in the South Indian population and discovered that the right femur's and left femur's transverse diameters were 42.65±3.25 mm and 42.36±3.69, respectively. Both the studies are almost by our study. Khaleel et al. [11] studied the proximal extremity of the femur and identified that the average transverse diameter of the head of the right femur was 37.86±3.06, and of the left femur it was 37.74±3.05; their values were slightly less than our study while Deshwal et al. [18] recorded values higher than our study. According to Ziylan et al. [9], the average transverse diameter of the head was 44.74.1 mm of the right femur and 44.33.3 mm of the left femur in the Anatolian population. The findings published by Ziylan et al. [9]. did not match the values found in our investigation, which may have been caused by variation in the population under study.

In this research, the mean values of the circumference of the femoral head on right and left-sided femur bones were 13.14± 0.49 and 13.38±13.38 cm, respectively, and their difference was identified to be statistically non-significant. The findings of our result are to the result of Rashid et al. [17], who found the mean circumference on the right side as 13.86±1.08 cm and on the left side as 13.71±1.13 cm. Osorio et al. [19], in their study on the circumference of the femoral head, found values of 14.49 ±0.94 cm on right and 14.17 ±1.56 cm on the left side, respectively, and their values were slightly higher than those of the present study while Dwivedi A et al. [14] discovered the circumference of head 126.50mm and 126.80mm of right and left femur respectively which are lower than our study.

This finding demonstrates that the mean vertical diameter of the right femur's neck was 28.45±4.22 mm, and of the left femur, it was 27.43±2.20 mm, and their difference was found to be statistically significant. Dwivedi et al. [14] prove that the mean vertical diameter of the neck of the right femur was 27.78±4.22 mm, and of the left femur was 27.43±2.20 mm is almost in concurrence with our study. Ziylan et al. [9] reported the morphometry of the Anatolian femur and quoted the result of vertical neck diameter on the right side as 30.6±3.0 and on the left side as 30.7±3.6 mm. Due to the variation in the population, Ziylan et al.’s results were contrasting with our study. Verma et al. [20] recorded the vertical diameter of the neck as 34.23 and 31.73 mm of the right and left femur, respectively, higher than our study. In males, neck thickness increases with an increase in age due to cam impingement which contributes to the development of osteoarthritis [19].

The difference between the transverse diameter of the neck of the femora on the right side was observed as 22.33±1.24 and on the left side was 23.25±3.22 mm respectively, and their difference was found to be statistically non-significant. Dwivedi et al. (2019) [14] found that the mean transverse diameter of the neck of the right femur was 23.28±2.85 mm, and of the left femur was 23.13±2.83 mm is almost in concurrence with our study. Our findings also correlate with Verma et al. (2017) [20]. Ziylan et al. (2002) [9] studied analysis of the Anatolian femur and reported that the values of the transverse diameter of the neck of the right femur were 25.5±2.7 mm, and of the left femur it was 26.3±3.1; the results obtained by Ziylan et al. [9] was contrasting to our results. This may be due to variations in the population studied [18].

The mean circumference of the neck of the femur on the right side was 9.73±7.93 cm, and that on the left side was 8.93±0.43 cm, and their difference was found to be statistically non-significant. Osorio et al. [19] found mean values of the circumference of the neck of the femur as 9.72 and 9.68 cm on the right and left side, respectively, and it is in concurrence with our study. Ziylan et al. [9] found higher values, while Khanal et al [21] recorded values lower than our study.

The mean anterior length of the neck of the femur on the right side was 2.86±0.36 cm, and that on the left side was 4.00±12.02 cm, and their difference was found to be statistically significant. Our study results agree with the studies of Dwivedi et al. [14] in the right femur, but we found a slightly higher value in the case of the left femur. The values obtained from the studies by Verma et al. [20], India, were more significant than our study. Ozandac et al. [22] recorded values lower than our study. Hence, it indicates that the values are different in different regions of India, which shows regional variations.

Caucasian postmenopausal women have a longer femoral neck length with femoral fractures when compared to women without fractures in a retrospective study conducted by El-Kaissai et al. [20]. According to Calis et al. [23] study on Turkish women, patients with hip fractures had considerably wider femoral necks and more acute angles. The femoral neck angle and neck length substantially correlate. Shorter people have more oblique femoral necks because their femurs are shorter, their femoral necks are shorter, and their neck-shaft angles are smaller. As a result, the taller person will have a more noticeable femoral neck [18].

In the present study, the mean neck-shaft angle of the right femur was 119.290±3.34 mm, and that of the left femur was 121.160±9.91 mm, and their difference was found to be statistically non-significant. The study done by Khan et al. [24] reported that the values of the neck-shaft angle of the right femur were 120±3.40 mm, and that of the left femur was 1240±4.2 mm, which is to our study. In the study, by Gujar et al. [25], the western population was a neck-shaft angle of 136.20, which was very high compared to other studies. Higher values were also noted by Rajendran et al. [26]. In a different investigation into a different population group, a wide range of changes in the neck-shaft angle was observed. The optimum neck-shaft angle is greater for urban residents than for non-mechanized rural residents [26,27].

Anderson et al. [28], in their study of 30 different population groups, stated that with the increasingly sedentary lifestyle, there is a noticeable trend towards higher mean angles. He observed that neck-shaft angle decreases in more heavy workers increase with decreasing mobility. According to Isaac et al. [29], forensic recognition of a person with pathological alterations who walks abnormally and has any suspected faulty angles may be useful. According to research by Toogood et al. [30], undertaken on 375 dry femur bones in the year 2009. There were numerous age-dependent variations in the femoral neck-shaft angle, in addition to variations in the FN version. These differences may be the supplementary reason for the rise in certain embryonic or developmental disorders [30].

At birth, the femur's femoral neck angle is zero, and it grows in tandem with the body's expansion. It reaches specific numbers at the age of eight and is directly relevant to the patient’s age and the length of the femur. Its growth is connected to verticalization and being able to Walk. Especially obvious examples of this are individuals with congenital hip dysplasia and other incidences of reduced or absent weight-bearing throughout growth [18].

The mean length of the intertrochanteric crest of the right femur was 5.92± 0.52 cm, and the left femur was 6.20±0.87 cm, and their difference was identified to be statistically significant. Akkaya et al. [31] suggested that the length of the intertrochanteric crest of the right femur was 67.5±4.9 mm, and of the left femur was 67.5±4.9 mm, which is almost to our findings. Khanal et al. [21] studied the measurements of proximal and distal fragments in the Nepalese population and found the length of the intertrochanteric crest of the right femur was 5.10 ±0.74 cm of the left femur was 4.98±0.66 cm which is lower than our study.

It's possible that geographic and racial variances are the cause of this discrepancy. During procedures for total hip replacement and femoral neck fracture, the greater and lesser trochanters are crucial landmarks in the circumferential placement of the femoral stem. Consequently, the lesser trochanter's apparent rotational fluctuations could lead to a number of positional mistakes. From this perspective, descriptors for this area that differ according to ethnicity, age, and gender have become more significant [30].

In our study, the dimensions of the proximal end of the right femur showed a highly significant positive linear correlation with the total length of the right femur. Among the dimensions, the vertical diameter of the neck of the right femur showed the highest degree of correlation with the length of the right femur (correlation coefficient 0.694). The study conducted by Dwivedi et al. on the-Maharashtrian population found a highly significant positive linear correlation of variables of the proximal end of the right femur with its total length. Among the dimensions, the vertical diameter of the head of the right femur showed the highest degree of correlation with the total length of the right femur (correlation coefficient 0.784).

In the present study, the proximal end of the left femora variables showed a highly significant positive linear correlation with the total length of the left femur. Circumference of the neck of the left femur showed the highest degree of correlation with the total length of the left femur (correlation coefficient 0.839). The study conducted by Diwedi et al. [14] on the Maharashtrian population found a highly significant positive linear correlation of dimensions of the proximal end of the right femur with its total length. Among the dimensions, the transverse head diameter of the left femur showed the highest degree of correlation with its total length (correlation coefficient 0.784) [32].

A measure of association between two variables is defined as the correlation [31]. The weight-bearing bones of the lower limb have the highest degree of correlation with stature [13] Chandran, in his study on South Indian females, concluded that all the incomplete measurements showed a positive correlation with the femoral length. Therefore, femoral length can be estimated from the fragmentary remains of the femur.108 In forensic anthropology, most researchers have used a degree of correlation for deriving regression equations to estimate stature, which is an essential parameter for medico-legal investigations as it helps identify missing persons [27,13].

The knowledge of morphometric measurements of femoral segments is helpful for orthopedics in the treatment of femoral fractures and may provide valuable guidelines to prosthetists and orthopedic surgeons to construct suitable implants. Various studies showed significant differences in the femurs of different gender, ages, race, and region. Therefore, statistical analysis of different parameters of the femur is indispensable.

Limitation of the study

Recent technologies are not used to measure femoral length only used traditional methods for calculating femur measurement.

## Conclusions

In the following study, the correlation of the maximum length of the femur with its proximal fragments was calculated on both the right and the left sides, and the maximum fragmentary measurements demonstrated positive correlations with the femoral length. Among the dimensions, the vertical diameter of the right femur’s neck showed the highest degree of correlation with the length of the right femur (correlation coefficient 0.694). Circumference of the neck of the left femur showed a greater degree of correlation with the total length of the left femur (correlation coefficient 0.839). The understanding of morphometric measurements of femoral segments aids orthopedics in the treatment of femoral fractures and may offer valuable standards to prosthetists and orthopedic surgeons in the construction of appropriate implants. It may also inspire biomechanical engineers to take a bold step forward in modifying implant designs to meet our needs. In medico-legal instances, assessing stature and recognizing unidentified bodies are also crucial. Different climatic circumstances, inherited traits, dietary habits, lifestyle choices, and other geographical features typically have an impact on the morphometric measures of the femur from various areas and countries. As a result, different populations' statistical analyses of the morphometry of the femur vary. Consequently, each individual bone needs to be the subject of a similar study.
